# *Bartonella henselae* and Domestic Cats, Jamaica

**DOI:** 10.3201/eid1107.050115

**Published:** 2005-07

**Authors:** Locksley L. McV. Messam, Rickie W. Kasten, Megan J. Ritchie, Bruno B. Chomel

**Affiliations:** * University of California, Davis, California, USA;; † Jamaica Society for Prevention of Cruelty to Animals, Kingston, Jamaica

**Keywords:** Bartonella henselae, Caribbean, cat scratch disease, domestic cats, Jamaica

**To the Editor:** *Bartonella henselae* has been isolated from domestic cats in most countries where it has been investigated (1), with the exception of some countries at northern latitudes, such as Norway (2). The prevalence of both bacteremia and seropositivity in cats is usually highest in warm and humid tropical countries. The worldwide distribution of cat scratch disease (CSD), a zoonotic disease caused mainly by the scratch of a *B. henselae*–infected cat, follows a similar pattern. Limited information is available about CSD in either humans or the feline reservoir in the Caribbean region.

In 1955, 3 febrile children (siblings) admitted to a hospital in Havana, Cuba, were diagnosed with CSD based on their symptoms and the positive results of intradermal tests using the Foshay antigen (3). The results of the bacteriologic examination, however, were negative. All 3 siblings had previous contact with a female cat and her 4 kittens. In 2003, Alvarez et al. (4) reported the case of a 13-year-old Cuban boy who was treated for symptoms compatible with CSD. However, no other information could be found in the scientific literature regarding the isolation of this bacterium from domestic cats in the Caribbean or seropositivity for *B. henselae* in humans or animals living in that region.

In the summer of 2003, an employee at a veterinary clinic in Kingston, Jamaica, was scratched and bitten on the hand by a cat. Fever and an enlarged axillary lymph node developed in the employee, and CSD was suspected. To confirm the clinical suspicion, and with the employee's permission, a serum sample was taken 7 weeks after the incident. Whole blood from the 62 remaining cats in the cattery was also collected into EDTA-containing tubes and stored at 4°C before being shipped to California for testing. The cat involved in the incident was not available for testing. The age of 63% of the cats ranged from 1 month to >5 years. Forty percent of the cats were formerly owned and put up for adoption and 16% of the cats were strays. The cat's origin was not recorded for the remaining 44% of the cats.

Upon reception at the laboratory, all cat blood samples were frozen at –70°C. They were subsequently thawed, and aliquot plated onto 5% rabbit blood–enriched agar and incubated at 37°C in 5% CO_2_ for ≤4 weeks. The EDTA tube supernatant was serogically tested for *B. henselae* (mixed type I and type II antigens) by using a standard indirect immunofluorescence assay (5). The 62 blood samples were cultured and 12 (19.3%) cats were bacteremic for *B. henselae*. None of the cultures yielded *B. clarridgeiae* or *B. koehlerae*. Of the 12 bacteremic cats, 5 (42%) had positive cultures for *B. henselae* type Houston I, and 7 (58%) had positive cultures for *B. henselae* type Marseille, based on restriction fragment length polymorphism profile of the 16S rDNA, by using *DdeI* enzyme (6). The median number of CFUs was 385/mL (range 147–25,300). For the 5 cats infected with *B. henselae* type Houston I, the median was 259 (range 147–513) CFU/mL; for the 7 cats infected with *B. henselae* type Marseille, the median was 534 (range 174–25,300) CFU/mL. Of the 5 cats that were bacteremic for *B. henselae* Houston I, 2 were seronegative. Similarly, 2 of the 7 *B. henselae* type Marseille–bacteremic cats were seronegative. These 4 seronegative cats, 4–10 weeks old, were most likely in the early phase of bacteremia. None of the cats were co-infected with both subtypes. When a titer of ≥1:64 was used, 37 (60%) cats were seropositive for *B. henselae*. Their age ranged from a few weeks to >5 years old (median 11 months), including 7 cats that were <6 months old. The employee's *B. henselae* titer was 1:64.

These results constitute the first report originating from the Caribbean region of *B. henselae* isolation from domestic cats, as well as confirming seropositivity in a human, despite a low titer. Because we were not able to obtain a blood sample from the suspect animal, we cannot prove that this cat was the source of the employee's infection. Nevertheless, this study confirms the existence of both *B. henselae* types I and II in Jamaica, even if no specific conclusions can be drawn with regard to their relative prevalence.

The Caribbean has the highest incidence of HIV/AIDS outside of sub-Saharan Africa, with Jamaica having a HIV prevalence of 1.2% (range 0.6%–2.2%) for persons 15–49 years of age (7). As *B. henselae* is known to cause bacillary angiomatosis and bacillary peliosis in immunocompromised persons, knowledge of its presence in the Jamaican cat population is important for primary prevention. Unfortunately, diagnostic tests for *B. henselae* are not currently available on the island.
